# The Combined Effect of Downregulated *RB1* and Overexpressed lncRNA SSTRS-AS1 on Prediction Time to Castration-Resistant Prostate Cancer: Indonesian Cohort Studies

**DOI:** 10.5152/tud.2022.21282

**Published:** 2022-03-01

**Authors:** Indrawarman Soerohardjo, Andy Zulfiqqar, Irianiwati Widodo, Didik Setyo Heriyanto, Sumadi Lukman Anwar

**Affiliations:** 1 Division of Urology, Dr. Sardjito General Hospital, Public Health and Nursing UGM Faculty of Medicine, Yogyakarta, Indonesia; 2 Department of Anatomical Pathology, Public Health and Nursing, Universitas Gadjah Mada Faculty of Medicine, Dr. Sardjito Hospital, Yogyakarta, Indonesia; 3 Division of Surgical Oncology, Department of Surgery, Public Health and Nursing, Universitas Gadjah Mada Faculty of Medicine, Dr. Sardjito Hospital, Yogyakarta, Indonesia

**Keywords:** ADT, biomarkers, CRPC, prostate cancer, RB1, SSTR5-AS1

## Abstract

Objective: Identifying the mechanism underlying the initiation and development of castration-resistant prostate cancer remains challenging. Time to castration-resistant prostate cancer is defined by prostate-­specific antigen progression and may represent a risk factor for developing immune alterations with a negative prognostic role in the overall survival of patients with prostate cancer. This study aimed to evaluate the combined effect of downregulated *RB1* and overexpressed SSTR5-AS1 as biomarkers for predicting time to castration-resistant prostate cancer.

Material and Methods: The clinical and pathological data of patients with prostate cancer were collected retrospectively. Between 2015 and 2019, a total of 36 patients who received castration were included. Expressions of mRNA of *RB1* and SSTR5-AS1 from primary tumors were quantified using quantitative real-time polymerase chain reaction. Patients were divided into 2 groups: the first group consisted of patients with *Rb1* expression lower than the median and expression of SSTRS5-AS1 higher than the median, and the second group consisted of all the other patients. This study was conducted in compliance with the latest Helsinki Declaration and registered on Elsevier International Standard Randomized Controlled trial number registry.

**Results: **In this study, patients with both downregulated RB1 and overexpressed Long non-coding RNAs (lncRNA) SSTR5-AS1 showed shorter time to castration-resistant prostate cancer (mean 23.6 ± 3.3 months) compared to other groups (mean 38.3 ± 4.9 months) (log-rank test, *P* = .028).

Conclusion: The combination of downregulation of RB1 and overexpression of SSTR5-AS1 is a strong predictor of shorter time to castration-resistant prostate cancer in the Indonesian population. Additionally, patients with International Society of Urological Pathology (ISUP) score >4 did not demonstrate this predictive value on time to castration-resistant prostate cancer.

**Main Points**

- The combination of low expression of RB1 and overexpression of lncRNA SSTRS-AS1 has prognostic value to predict the outcome of castration therapy in the Asian population.
- There is no difference in castration methods on the term time to castration-resistant prostate cancer.
- Disease burden classified based on Chemohormonal Theraphy versus Androgen Ablation randomized Trial for Extensived Disease In Prostate Cancer (CHAARTED) study has no prediction value to predict the outcome of castration therapy.

## Introduction

Prostate cancer is recognized as the malignancy that has the second-highest incidence in men with an annual incidence of 1.3 million in 2018.^1^ The carcinogenesis of prostate cancer is specifically influenced by the hormonal milieu, which includes androgen.^2^ The hormonal therapy known as castration therapy was started back in 1941 when American surgeons found that endocrine manipulation was followed by regression in prostate cancer. Since then castration and its axis have become pillars for managing advanced local and metastatic prostate cancer.^2-4^ Despite successful responses in many patients with prostate cancer toward castration, the majority of treated subjects eventually will develop the resistant castration variant of prostate cancer, and this condition is known as castration-resistant prostate cancer (CRPC). The median time to CRPC on metastatic population in Indonesia has been reported approximately as 24 months.^5^

Castration-resistant prostate cancer is defined as the progression of prostate cancer despite hormonal manipulation that is marked as testosterone value <50 ng/mL. The progression may show as clinical progress, imaging progression, or increment of serum prostate-specific cancer antigen (PSA) value.^6^ Because patients receiving androgen deprivation therapy (ADT) often may no longer respond to castration therapy and the time to develop CRPC varies between patients, biomarkers to guide the clinical decision-making are essential for managing this cancer.^7,8^

Novel treatment and biomarker modalities need to be developed, and significant efforts are being implemented with advances in progress being reached in basic, translational, and clinical research fields. However, the diagnostic approaches are still limited, particularly in advanced stages of the disease, due to the heterogeneity and complexity of the disease. Hence, specific biomarkers are needed to help in clinical settings to identify early responses to treatment outcomes and to identify the population of patients who are most likely to benefit from the ADT treatment.^6^

It is worth noting that prostate cancer has a marked endocrine nature with other non-sex hormones such as somatostatins,^1-5^ which are also related to normal prostate and prostate cancer development. Several studies have reported the role of somatostatin receptor (SSTR) signaling pathway in different types of tumors with SSRT5 expression being more predominant in all tissue samples.^7-9^ Furthermore, the antisense RNA 1 of SSTR5 (SSTR5-AS1) has been reported to have a crucial role to initiate cytotoxic or cytostatic antiproliferative signals in the induction of RB1 and G1 cell cycle arrest that leads to neuro-endocrine prostate cancer (NEPC) or CRPC.^9^ Thus, this study aimed to evaluate the potential clinical role of the combination of *RB1* and SSTR5 to predict the outcome of castration therapy in an Indonesian population from the Javanese ethnic group. 

## Material and Methods

### Patients

A total of 36 patients with prostate cancer and patients with benign prostatic hyperplasia (BPH) as control arms who underwent prostate biopsies between 2015 and 2019 at our hospital were enrolled. All patients who received castration as single therapy were enrolled in this study. Patients who received chemotherapy, immunotherapy, and local therapy were excluded from this study. This study received approval from the Medical and Health Research Ethics Committee (KE/0158/02/2020). 

The primary outcome of this pilot study was response to therapy toward castration that is described as time to achieve resistance toward castration. This condition is defined as the clinical progress or radiographic progression or/and increase in PSA values during hormonal therapy after achieving nadir values with testosterone level <20 ng/mL. Clinical staging was determined by unified tumor, node, and metastases criteria according to TNM eighth edition, 2017.^6^ The staging was determined by digital rectal examination, magnetic resonance imaging, computed tomography with contrast, or bone survey.

### Quantitative Real-Time Polymerase Chain Reaction

Following the published methods previously described by Soerohardjo et al^1^ this study was conducted in compliance with the most recent Helsinki Declaration, and the data were shared as a study registered with the International Standard Randomized Controlled trial number registry under reference 24834343.

RNAs were extracted from formalin-fixed and paraffin-embedded biopsied samples of prostate cancer tissues and 2 additional BPH tissues which were used as normal references. Hybrid-R™ Isolation Kit (GeneAll, Seoul, South Korea) was used to extract total RNAs, and NEXpro™ qRT-PCR Kit (NextPro, Seoul, South Korea) was used to measure SSRT5-AS1 expressions. Primer sequences that we used on this study were: 5ʹ-GACCCAGAAGCCATTGAAATCT-3ʹ (forward) and 5ʹ-GGTGTGCTGGAAAAGGGTCC-3ʹ (reverse) for RB1 with 5ʹ GCGTGTTTGTGCCTGTCCTG-3ʹ (forward), and 5ʹ-ACTACAGGTGCCATCAGACC-3ʹ (forward) and 5ʹ-GGTGTGCTGGAAAAGGGTCC-3ʹ (reverse) for SSRT5-AS1. The amplification conditions consisted of an initial denaturation step at 95˚C for 10 minutes, followed by 40 cycles at 95˚C for 20 seconds, at 55˚C for 40 seconds, and at 72˚C for 60 seconds. An extension was done at 7v2˚C for 5 minutes. The quantitative polymerase chain reaction-amplified samples were analyzed using BiONEERExi cycle™ 96 (BioNEER, Daejeon, South Korea). Low expression was described as the expression of both RB1 and SSTRS-AS1 below the median, and the high expression was described as expression of both RB1 and SSTRS-AS1 above the median. The first group in this study consisted of patients with low expression of *RB1* and high expression of SSTRS-AS1, and the second group included all patients who were not included in the first group. 

## Results

In this study, the mean of patients’ ages was 69.07 ± 8.7 years, while the mean of PSA at diagnosis was 141.22 ± 112.28 ng/mL. Included subjects were predominately patients in the ISUP group 5 (47.2%), and the castration method was performed using Luteinizing Hormone-Releasing Hormone (LHRH) agonists (55.6%). The majority of patients have comorbidities, which were dyslipidemia (44.4%) and type 2 diabetes mellitus (36.1%) (Table 1).

**Figure 1.**
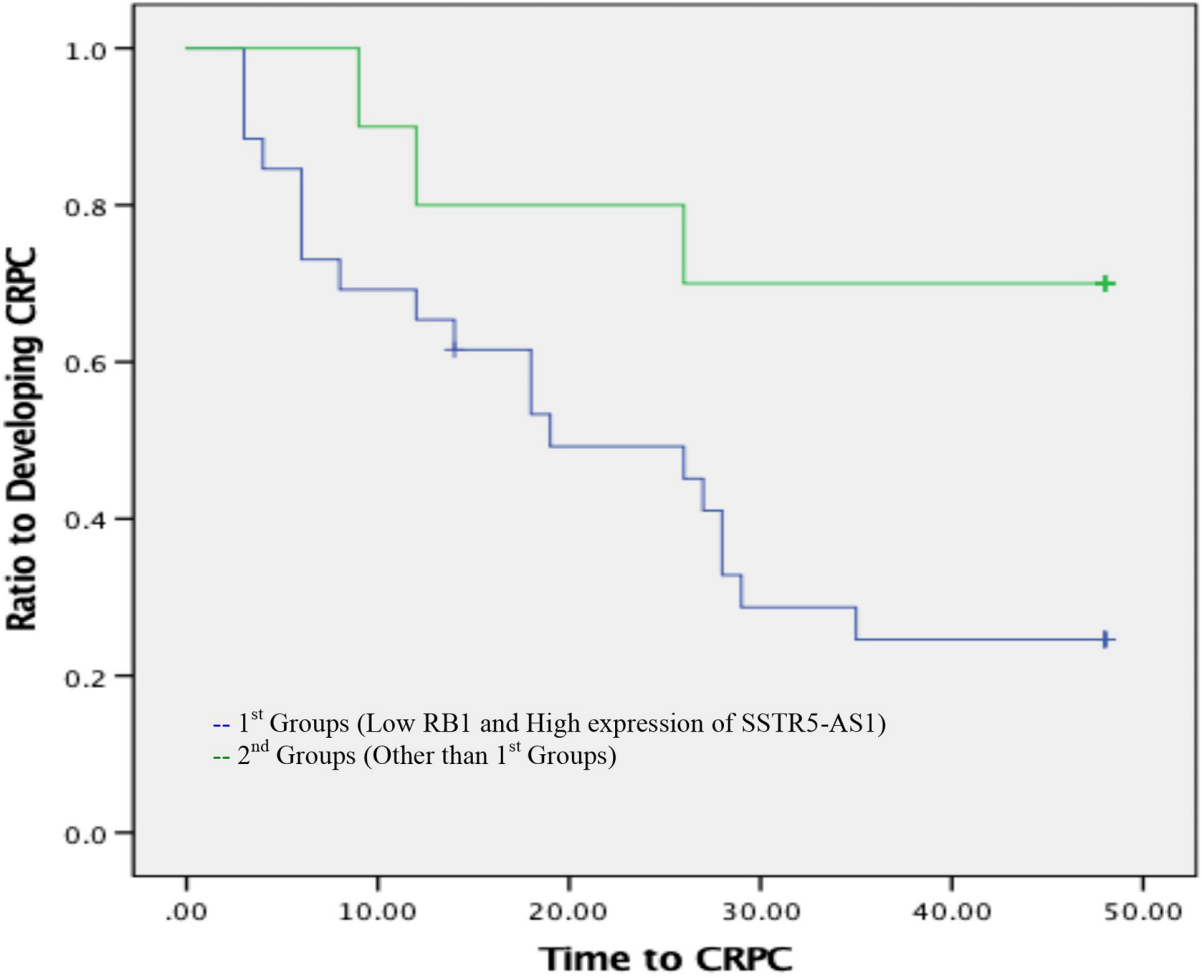
The expressions of SSTR5-AS1 (left) (*P* > .05) and Rb1 (right) (*P* < .001) on prostate cancer with no metastases and bone metastases at time of diagnoses (*P* < .001).

The expression of SSTR5-AS1 was higher in non-metastatic patients compared to patients diagnosed with metastatic diseases at initial stages, but there was no statistical significances. However, the expression of RB1 was significantly higher in non-metastatic diseases group (Figure 1).

**Figure 2.**
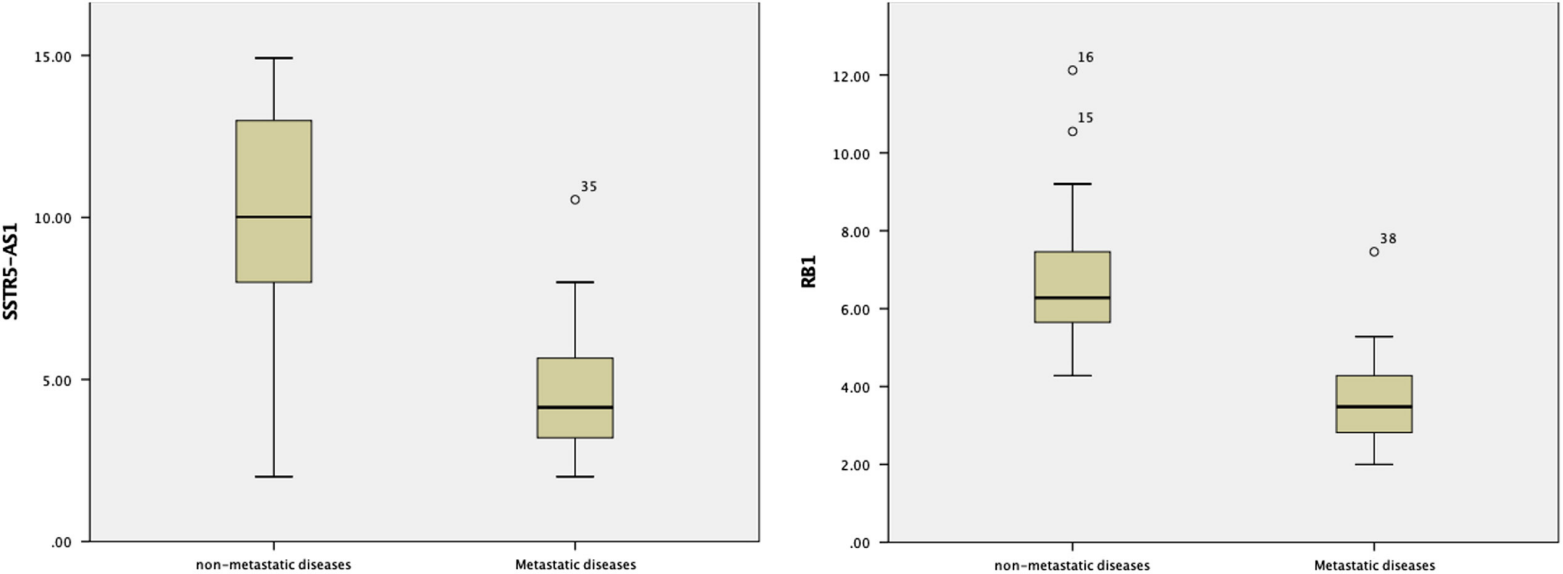
Kaplan–Meier estimates of time to castration-resistant prostate cancer in prostatic cancer patients who received androgen deprivation therapy as therapy of prostate cancer for expressions of RB1 and SSTR15-AS1.

The patients with low RB1 expression and high expression of SSTR5-AS1, which is described as the first arm, have a shorter time to CRPC (mean 23.6 ± 3.3 months) compared to other patients (mean 38.3 ± 4.9 months) (Figure 2). In comparison tests, this difference was statistically significantly different (log-rank test, *P* = .028). Meanwhile, the castration approaches, the diseases burden according to the CHAARTED study, and the value of PSA >20 did not show statistical significance to predict time to CRPC (Table 2).

## Discussion

The overexpression of SSTRs is commonly found in neuroendocrine tumors (NED) and treatment-induced NEPC. The expression has been correlated with the worse prognosis in many types of solid cancers due to limited treatment options.^9-12^ In order to function biologically and activate its signaling pathway, SSRTs need to bind with 5 family gene products (SSTR1-SSTR5).^11,12^ The development of both NED and the overexpression of SSTRs have been associated with a worse prognosis, which is a shorter time to CRPC.^13,14^ Although, the context of NED in prostate cancer is controversial due to the unknown mechanism by which it develops and the limited clinical significance, yet, some studies have reported the presence of neuroendocrine cells in prostate cancer.^12,13,15^

The RB1 itself is known for its role in regulating cell cycle progression, which is recognized in its clinical impact for treatment that is focused on specifically targeting cyclin-dependent kinase 4/6 inhibitor pathways.^16,17^ Clinically, the use of *RB1* as a biomarker for predicting treatment outcome has shown promising results. The downregulation of *RB1* or loss of expression of the protein level have shown worse outcomes in castration therapy.^1^

As far as we know, this study is the first research in an Indonesian population to evaluate the combination of the *RB1* and SSTRS-AS1 biomarkers. This study has shown that the combined effect of low expression of RB1 and high expression SSTRS-AS1 provides promising parameters for predicting worse outcomes of castration as a single therapy in prostate cancer. This result may indicate that patients with high expression of RB1 and low expression of SSTRS-AS1 are the most beneficial population to receive castration as their single therapy compared to other populations. We found evidence to support routinely evaluating these biomarkers in patients who underwent upfront, neoadjuvant chemotherapy. 

The function of SSTRS-AS1 has not been fully characterized, but its overexpression is known as a biomarker in the identification of NEPC.^18^ Recently, one study prospectively evaluated SSTR-targeted therapies using somatuline autogel in the phase IV CALMNET trial (NCT02075606), although the final results have not been published yet. These biomarkers not only have potential predictive value for the outcome of castration therapy but also may change the paradigms on managing prostate cancer from the sequence line of therapy into the more selective therapy. 

In our previous study, *RB1* showed no statistical significance for predicting time to CRPC. This finding reflects its role after we analyzed the expression according to the distant metastatic results.^1^ Another study previously conducted by Hamid et al^19^ also found that alterations of *RB1* expression are not significant when analyzing the combined effect of several tumor suppressor genes:* RB1*, *TP53*, and *PTEN*.

One limitation of this study was the sample size as only limited number of patients were enrolled. Further research is needed with more samples and longer observation times including follow-up of the outcomes of patients who received either upfront chemotherapy or chemotherapy as second-line therapy after developing CRPC. One of the strengths of this study was the racial homogeneity of the samples enrolled, which only included subjects from the Indonesian population who originated from the island of Java in Indonesia.

## Figures and Tables

**Table 1.** Demographic Characteristics

Variables	
Age (years ± SD 95%)	69.07 ± 8.7
PSA	141.22 ± 112.28
ISUP groups (%)	
1	5 (13.9%)
2	4 (11.1%)
3	1 (2.8%)
4	9 (25%)
5	17 (47.2%)
Surgical castration (%)	
Yes	16 (44.4%)
No	20 (55.6%)
T staging (%)	
T1a	4 (11.1%)
T1b	2 (5.6%)
T1c	9 (25%)
T2a	2 (5.6%)
T2b	10 (27.8%)
T2C	7 (19.4%)
T3C	2 (5.6%)
N staging (%)	
Nx	29 (80.6%)
N0	4 (11.1%)
N1	3 (8.4%)
M staging (%)	
M0	18 (50%)
M1B	18 (50%)
Comorbid (%)	
Cerebrovascular	10 (27.8%)
Dyslipidemia	16 (44.4%)
ESRD	7 (19.4%)
T2DM	13 (36.1%)

PSA, prostrate specific antigen; T2DM, type 2 diabetes mellitus; ESRD, End-Stage Renal Disease.

**Table 2.** Predictive Value Time to CRPC

Variable	Time to CRPC (Months)	*P*
First group (low RB, high SSTRS-AS1)	23.6 ± 3.3	.028
Second group (high RB, low SSTRS-AS1)	38.3 ± 4.9
Surgical castration	32.12 ± 3.9	.132
Medical castration	22.63 ± 4.17
High volume sisease	26.75 ± 3.48	.728
Low volume disease	28.67 ± 4.7
PSA > 20 ng/mL	24.85 ± 3.13	.072
PSA < 20 ng/mL	37.88 ± 6.25

*P *values were calculated using log-rank test.

CRPC, castration-resistant prostate cancer; PSA, prostrate-specific antigen.
